# Supplementary material for: Nonoverlap proportion and the representation of point-biserial variation
Source: PLoS One. 2020 Dec 28;15(12):e0244517. doi: 10.1371/journal.pone.0244517 (PMC7769483; doi:10.1371/journal.pone.0244517)
Supplement: Attachment — Submitted filename: ResponseToReviewers.pdf [file pone.0244517.s002.pdf]

November 10, 2020

Nonoverlap proportion and representation of point-biserial variation

PONE-D-20-22303

Response to reviewers

I would like to thank the Reviewers for their very helpful comments, and the opportunity to make this revision. The Introduction has been re-written to emphasize the importance of constructing a self-consistent algebraic framework for point-biserial variation. Section 1.2 has also been revised to more clearly describe our solution for this problem. I believe that the presentation has been significantly improved by these changes, and I hope there is less confusion. My responses to the Reviewers comments are highlighted in blue.

Stanley Luck

Reviewer #1

Reviewer's comments: The paper especially the introductions and abstract are hard to read and follow which may due to the different established stats concepts mixed together, and these in general are applied to the different scenarios.

My remarks below explain why I believe that this assessment is incorrect.

The method section is too long, it's unclear what the original work are, and what established techniques are discussed in the paper. This paper may be more suitable to submit the statistical types of journals based on the method section.

I believe that this work is timely and addresses central problems in applied statistics with implications for a wide range of applications. PLOS ONE has a multidisciplinary audience and provides open access, and is therefore my preferred choice for publication.

The following are some detailed comments:

1. Title: is not informative, with only include two statistical terms.

- 'Representation' and 'variation' are important operative terms, from vector algebra and applied math.
- Nonoverlap proportion is an important parameter in the representation of point-biserial variation.
- 'Proportion' refers to the $\mathbf{c_y}$ sorting algorithm and the corresponding 2×2 contingency table.
- 'Point-biserial variation' refers to the parameterization of statistical variation for two sets of $\mathbb{R}^1$ data.
- 'Point-biserial' indicates that this is effect size research.

2. Abstract: There are many notations in the abstract which have been messed up due to the formatting, and made the paper very hard to read and follow, e.g., what do R2, P1, etc. represent; why author choose to use rpb, ppb, rpbd types of abbreviation for the key terms of the paper.

'Point-biserial' and r_{pb} are established terminology in the effect size literature, as acknowledgment of Karl Pearson's seminal contribution. The notation r_c has been used previously, but I prefer r_{pbd} because it emphasizes the algebraic connection with r_{pb}. Then, the ρ_{pb} notation seems appropriate in order to be consistent.

Readers will lose interests if contents re too technical in the abstract. It should start with problem description or issues and challenges need be addressed, and then provide statistical solution with applications. The author may simplify the intro section and state clearly what the new work is proposed in this paper, and what are the associated applications.

The Introduction has been re-written to more clearly describe the objectives, and the Methods has been revised as well.

3. Keywords, too many should limited to 5-6.

Note that Reviewer #2 has pointed out that additional keywords are needed.

4. Introduction: page 2, author started with the Pearson product moment correlation coefficient (r) and δ (mean differences, the numerator of Cohen's d), note that Pearson r and Cohen's d are two well established stats concepts, each with specific formula to compute the effect size measures. They are applied for two different scenarios: the former is used to correlated two continuous variables (such as age and BMI); while the latter is used for one continuous and one binary (such as binary treatment (y/n) on blood pressure). It's unclear what are the motivation to mix two scenarios and build the connection between these different effect size measures. It's inappropriate to simply apply Pearson r in the case of one binary and one continuous variable situation which is why there are list of difficulties author mentioned from line 19-24. If the author intends to correlate the paired data such as before and after, he may need make it clear.

- The fact that the study of biserial correlation was initiated by Karl Pearson speaks for itself.
- Consider the well-known relation between t and r_{pb}

  $$t = (N-2)\frac{r_{\text{pb}}}{\sqrt{1-r_{\text{pb}}^2}},$$

  (Eq 4, Kornbrot, 2005, `https://onlinelibrary.wiley.com/doi/pdf/10.1002/9781118445112.stat06227`). Aside from the $(N-2)$ scaling factor, t^2 is a linear fractional transformation of r_{pb}^2. Thus, t serves as a

measure of point-biserial association, and in rCART association graphs, t (not shown) and r_{pb} give equivalent results.

Now, conjecture that there is a 'mixing up of scenarios' so that r_{pb} is an undefined quantity, and therefore a meaningless construct. Then, t and r_{pb} are both undefined. Similarly, we regard r_{pb}^2 as corresponding to a linear fractional transformation of $p_{\text{A}}p_{\text{B}}d^2$ (see Eq 2 and Fig 3), and we conclude that either d or $p_{\text{A}}p_{\text{B}}$, or both are undefined. Then, we are not able to do statistics because $\mathbf{v}_{\text{pb}}$ is undefined, which is a contradiction. Therefore, we conclude that the 'mixing up of scenarios' conjecture is false.

- In this paper, I consider the problem of constructing a self-consistent algebraic framework for point-biserial variation, as described in the Introduction. Using NHC data, I also provide a realistic demonstration of why the resolution of the 'mixing up of scenarios' issue is important. The status quo with the lack of consensus on the merits of r_{pb} creates confusion, hindering the application of effect size in data analytics.

5. Line 16-17, p value answers hypothesis testing question of statistical significance, while effect size measure is related to estimation question, they are different stats techniques, and serves for different purpose, so they are not alternative. It's true that p value has the drawback of sample size dependence, and the effect size shows the clinical/practical relevance/importance regardless of sample size.

- The confidence interval for effect size will be sample size dependent.

- The algebraic relation with r_{pb} raises the question of what exactly is the distinction between effect size and t-test methodology. This is a topic for a separate paper.

- There is an equivalence:

$$t \leftrightarrow (t, a) \leftrightarrow \left(r_t, \sqrt{1 - r_t^2}\right),$$

  where $r_t = t/\sqrt{t^2 + a^2}$, and $a = 1$ is the default choice. Similarly, there is an equivalence $d \leftrightarrow r_{\text{pbd}}$. There are different coordinate systems for the representation of effect size; i.e. system response. The $\mathbb{S}^1_+$ representation, $\{r_t, r_{\text{pbd}}\}$, is convenient in data analytics.

6. Line 24-25: "dichotomy of a normal distribution", do you mean bivariate normal?

The line of research in Gradstein's paper involves the dichotomy of a normal distribution, which derives from Pearson's biserial statistics research.

7. Line 24 What does this mean "In Paper1", is this published?

Line 13, revised manuscript.

8. Line 24, "2×2 contingency tables" refer to two binary variables situations? Why is here/

See Eq 8.

9. It's known taking the square the Pearson r value, one can obtain R^2, which shows the variations shared by 2 continuous variables, if R^2 subtracted by 1, one can get the no-overlap portion not shared by two variables,

- Note that r_{pb} is already a measure of nonoverlap because $r_{\text{pb}} \to 1$ as Cohen's $d \to \infty$, as shown in Fig 2.
- Together, R^2 and your $1 - R^2$ correspond to a point, $(R^2, 1 - R^2) \in \triangle^1$, in the standard one-simplex with one-degree of freedom. Then, one

parameter is sufficient to represent the variation. Therefore, $(1-R^2)$ does not provide any new information.

- For uniform and normal distributions, we can obtain algebraic expressions that relate r_{pbd} and ρ_{pb}. However, notice that $r_{\mathrm{pbd}} = \rho_{\mathrm{pb}} = 0$, and $r_{\mathrm{pbd}} = \rho_{\mathrm{pb}} = 1$ are both allowed. Therefore, for arbitrary distributions there is no general constraint that connects r_{pbd} and ρ_{pb}. r_{pbd} and ρ_{pb} are different parameters (see Eq 18) for point-biserial variation as demonstrated in section 2.

why your proposed novel "point-biserial variation" is needed?

- See Eq 8 & 18. The rigorous formulation of effect size requires the specification of a complete set of parameters for point-biserial variation, $\mathbf{v}_{\mathrm{pb}}$.
- Then, the algebra for effect size is associated with the projective geometry for $\mathbf{v}_{\mathrm{pb}}$. Without the $\mathbf{v}_{\mathrm{pb}}$ specification, it will be difficult to resolve questions about the interpretation of effect size.
- I demonstrate that three parameters, $(r_{\mathrm{pbd}}, \mu, \rho_{\mathrm{pb}})$, are needed to distinguish different forms of point-biserial variation. See line 355.
- Cohen's d is a perspective function of $\mathbf{v}_{\mathrm{pb}}$ and provides an incomplete representation of point-biserial variation. Therefore, as a standalone measure d is subject to irreproducibility.

10. Line 48, "paper1" mentioned second time, it appears without reading author's "paper 1", it's difficult to connect and understand the techniques discussed here.

- In Paper1, I provide a rigorous framework for the analysis of a 2×2 contingency table and give a detailed explanation of why the ϕ coefficient should

be abandoned. ϕ is widely used as a measure of linkage disequilibrium in GWAS.

- ϕ and r_{pb} are both variants of Pearson r.
- Like r_{pb}, the confounding properties of ϕ have been studied by many researchers and their work is largely ignored.

11. Method: it's unclear what are the new/original work, and what are old/established techniques discussed here. Too long and hard to read.

- In Methods, I develop the mathematical foundations for point-biserial data analysis. The main novel contributions are already listed in the Introduction. See Eq 4, 8, 9, 10, 11, 15, 16, 18, 20, and Table 1.
- Old: Pearson $r(\mathbf{x}, \mathbf{y})$ is the standard measure of correlation. Pearson r is most useful when the data are correlated, $r \approx 1$, then $\mathbf{x}$ and $\mathbf{y}$ are equivalent, or nearly so (line 288). r is not very informative when $r \ll 1$.
- New: In Paper1, I demonstrate that the CART algorithm can be viewed as an exhaustive search over 'association graphs'. An association graph is an alternative to statistical association,

$$r \to \text{rCARTgraph},$$
$$\{t, d\} \to \text{bCARTgraph}.$$

- As described in section 1.5, the rCART association graph is a new method for analyzing association/dependence for $(\mathbf{x}, \mathbf{y})$ data that are not well correlated (line 291). This includes data for population studies where the system performance is determined by trade-offs between multiple factors. The applications for CART decision tree methodology include GWAS, the

assessment of nursing home performance, and other high-dimensional data analytics problems.

- Using the $\mathbf{v}_{pb}$ parameterization to obtain more functionally relevant classifications compared to IG_{MSE}, constitutes a significant change in regression tree methodology. The full implication of this is a topic for another paper.

12. It was poorly formatted, mixed with single spaced with double spaced. Figure' title is separated from figure.

The format of the PLOS draft document is different from the one that I originally submitted. The layout of my draft has already been checked for compliance with PLOS requirements.

Reviewer #2

1. There is no expansion for CART but the abbreviation is frequently used.

Line 3, revised manuscript.

2. Proper citation to be used for paper 1

Line 14, revised manuscript.

3. Keywords are missing

The new keywords are: classification imbalance, machine learning.

4. Proposed work explanation is not clear, So should explain clearly.

The Introduction has been re-written, and objectives are more clearly explained in Methods.

5. Need to check Spacing and Alignment of the paper

The format PLOS draft document is different from the one that I originally submitted. The layout of my draft has already been checked for compliance with PLOS requirements.

6. Add recent references.

Line 49, revised manuscript, includes a reference for the Johnson and Khoshgoftaar (2019) paper.
