# Supplementary material for: Nonoverlap proportion and the representation of point-biserial variation
Source: PLoS One. 2020 Dec 28;15(12):e0244517. doi: 10.1371/journal.pone.0244517 (PMC7769483; doi:10.1371/journal.pone.0244517)
Supplement: Attachment — Submitted filename: PLOS one revew.docx [file pone.0244517.s001.docx]

Reviewer’s comments:

The paper especially the introductions and abstract are hard to read and follow which may due to the different established stats concepts mixed together, and these in general are applied to the different scenarios. The method section is too long, it’s unclear what the original work are, and what established techniques are discussed in the paper. This paper may be more suitable to submit the statistical types of journals based on the method section. The following are some detailed comments:

1. Title: is not informative, with only include two statistical terms.
2. Abstract: There are many notations in the abstract which have been messed up due to the formatting, and made the paper very hard to read and follow, e.g., what do R2, P1, etc. represent; why author choose to use rpb, ppb, rpbd types of abbreviation for the key terms of the paper. Readers will lose interests if contents re too technical in the abstract.

It should start with problem description or issues and challenges need be addressed, and then provide statistical solution with applications. The author may simplify the intro section and state clearly what the new work is proposed in this paper, and what are the associated applications.

3. Keywords, to many should limited to 5-6.
4. Introduction: page 2, author started with the Pearson product moment correlation coefficient (r) and δ (mean differences, the numerator of Cohen’s d), note that Pearson r and Cohen’s d are two well established stats concepts, each with specific formula to compute the effect size measures. They are applied for two different scenarios: the former is used to correlated two continuous variables (such as age and BMI); while the latter is used for one continuous and one binary (such as binary treatment (y/n) on blood pressure). It’s unclear what are the motivation to mix two scenarios and build the connection between these different effect size measures. It’s inappropriate to simply apply Pearson r in the case of one binary and one continuous variable situation which is why there are list of difficulties author mentioned from line 19-24. If the author intends to correlate the paired data such as before and after, he may need make it clear.
5. Line 16-17, p value answers hypothesis testing question of statistical significance, while effect size measure is related to estimation question, they are different stats techniques, and serves for different purpose, so they are not alternative. It’s true that p value has the drawback of sample size dependence, and the effect size shows the clinical/practical relevance/importance regardless of sample size.
6. Line 24-25:” dichotomy of a normal distribution”, do you mean bivariate normal?
7. Line 24 What does this means “In Paper1, “, is this published?
8. Line 24, “2 × 2 34 contingency tables” refer to two binary variables situations? Why is here/
9. It’s known taking the square the Pearson r value, one can obtain R^2, which shows the variations shared by 2 continuous variables, if R^2 subtracted by 1, one can get the no-overlap portion not shared by two variables, why your proposed novel “point-biserial variation” is needed?
10. Line 48, “paper1” mentioned second time, it appears without reading author’s “paper 1”, it’s difficult to connect and understand the techniques discussed here.
11. Method: it’s unclear what are the new/original work, and what are old/established techniques discussed here. Too long and hard to read.
12. It was poorly formatted, mixed with single spaced with double spaced. Figure’ title is separated from figure.
